# Prediction of Paroxysmal Atrial Fibrillation From Complexity Analysis of the Sinus Rhythm ECG: A Retrospective Case/Control Pilot Study

**DOI:** 10.3389/fphys.2021.570705

**Published:** 2021-02-19

**Authors:** Vadim Alexeenko, Philippa J. Howlett, James A. Fraser, Daniel Abasolo, Thang S. Han, David S. Fluck, Christopher H. Fry, Rita I. Jabr

**Affiliations:** ^1^Department of Biochemical Sciences, Faculty of Health and Medical Sciences, School of Biosciences and Medicine, University of Surrey, Surrey, United Kingdom; ^2^Department of Physiology, Faculty of Biology, Development and Neuroscience, University of Cambridge, Cambridge, United Kingdom; ^3^Centre for Biomedical Engineering, Faculty of Engineering and Physical Sciences, University of Surrey, Surrey, United Kingdom; ^4^Department of Diabetes and Endocrinology, Ashford and St Peter’s Hospitals NHS Foundation Trust, Ashford, United Kingdom; ^5^Department of Cardiology, Ashford and St Peter’s Hospitals NHS Foundation Trust, Ashford, United Kingdom; ^6^School of Physiology, Pharmacology and Neuroscience, Faculty of Biomedical Sciences, University of Bristol, Bristol, United Kingdom

**Keywords:** paroxysmal atrial fibrillation, ECG, Lempel–Ziv complexity, ROC analysis, ECG complexity indices

## Abstract

Paroxysmal atrial fibrillation (PAF) is the most common cardiac arrhythmia, conveying a stroke risk comparable to persistent AF. It poses a significant diagnostic challenge given its intermittency and potential brevity, and absence of symptoms in most patients. This pilot study introduces a novel biomarker for early PAF detection, based upon analysis of sinus rhythm ECG waveform complexity. Sinus rhythm ECG recordings were made from 52 patients with (*n* = 28) or without (*n* = 24) a subsequent diagnosis of PAF. Subjects used a handheld ECG monitor to record 28-second periods, twice-daily for at least 3 weeks. Two independent ECG complexity indices were calculated using a Lempel-Ziv algorithm: R-wave interval variability (beat detection, BD) and complexity of the entire ECG waveform (threshold crossing, TC). TC, but not BD, complexity scores were significantly greater in PAF patients, but TC complexity alone did not identify satisfactorily individual PAF cases. However, a composite complexity score (*h*-score) based on within-patient BD and TC variability scores was devised. The *h*-score allowed correct identification of PAF patients with 85% sensitivity and 83% specificity. This powerful but simple approach to identify PAF sufferers from analysis of brief periods of sinus-rhythm ECGs using hand-held monitors should enable easy and low-cost screening for PAF with the potential to reduce stroke occurrence.

## Introduction

Atrial fibrillation (AF) is the most frequently encountered sustained cardiac arrhythmia, affecting about 2% of the population. Its prevalence increases with age, rising to 10% of those aged over 80 years. Moreover, AF is associated with an acceleration of cognitive decline and risk of dementia (Singh-Manoux et al., 2017). It is also associated with a fivefold increased risk of ischaemic stroke, as well as increased stroke severity, mortality and disability relative to those arising from other causes (Dulli et al., 2003). Moreover, patients suffering a recurrent stroke are almost twice as likely to have identifiable AF as those presenting with a primary stroke (30 vs 17%; Han et al., 2018), although reporting rates are likely underestimated (Jorfida et al., 2016). In consequence, many patients discharged after a primary stroke are not prescribed anticoagulants, but general prophylactic use of anticoagulants in the absence of an AF diagnosis is not beneficial (Hart et al., 2018). Currently, AF is detected by continuous or periodic electrocardiographic monitoring over extended periods (Kirchhof et al., 2016), using invasive or non-invasive methods (Seet et al., 2011), which can be costly and require patient co-operation.

Paroxysmal AF (PAF) is a self-terminating condition with episodes lasting minutes to days and accounts for 25–60% of diagnosed AF cases (Seet et al., 2011). Studies indicate that stroke incidence is similar in patients with PAF or sustained AF (Banerjee et al., 2013), however, other studies differ (Takabayashi et al., 2015; Ganesan et al., 2016). Nonetheless, PAF is more difficult to detect, and when episodes do occur, up to 90% of those affected have no symptoms (Page et al., 1994), also risking a greater incidence of associated stroke and thromboembolism (Hart et al., 2007). There is therefore an unmet need to improve PAF detection using a non-invasive, low-cost method that could be used by a greater number of people.

Atrial fibrillation is associated with electrical and structural myocardial remodeling and autonomic dysregulation of the heart (Andrade et al., 2014; Nattel and Harada, 2014) which should be reflected in increased electrocardiogram (ECG) signal variability. However, changes to ECG characteristics, such as P wave morphology or heart rate variation, are generally poorly associated with AF incidence and consequent stroke, especially for prediction of PAF (Schaefer et al., 2014; Maheshwari et al., 2019). However, P-wave axis variation is a reasonable predictor (Maheshwari et al., 2019) and supports the concept that small variations of the sinus-rhythm ECG waveform might be useful to predict PAF. A recent study based on machine-learning systems used sinus rhythm ECG traces to extract an AF-signature algorithm with specificity and sensitivity of around 0.8 (Attia et al., 2019), providing further evidence that sinus rhythm ECGs may contain subclinical signs of AF. However, such an approach is computationally complex and does not provide information about specific ECG changes that correlate with AF. The present work develops a method based on analysis of sinus rhythm ECG trace complexity and its day-to-day variability. It offers a simpler tool to screen for PAF and as a novel metric it should also provide additional information that could be combined with other approaches.

Non-linear analytical methods are sensitive tools to estimate the irregularity of biomedical signals and have been used on electroencephalogram recordings to identify onset of epileptic seizures, or risk of Alzheimer’s disease (Hornero et al., 2009; Aarabi and He, 2017). The Lempel-Ziv algorithm (Lempel and Ziv, 1976; Kaspar and Schuster, 1987) complexity measure is widely used to estimate the entropy density of symbolic strings by analyzing the generation rate of new patterns. It has been widely used to analyze a variety of biological signals, including neuronal spiking (Amigo et al., 2004), the electroencephalogram (Abásalo et al., 2015) and human motion (Peng et al., 2014), and was also proposed as a feasible tool to assess the signal quality of the ECG (Zhang et al., 2016). The inherently chaotic nature of the ECG signal in both healthy hearts (Goldberger, 1991; Glass, 2009; Shaffer and Ginsberg, 2017) and during atrial fibrillation (Qu, 2011; Aronis et al., 2018) suggested the possibility to use such an estimator for diagnostic purposes. We have used this approach in a pilot study to combine two independent parameters of continuous sinus-rhythm ECG waveforms: day-to-day variabilities of overall signal complexity and also the R-R interval. We demonstrate that PAF prediction is possible with very high specificity and selectivity from recordings made with a simple hand-help device.

## Materials and Methods

### Study Design

Participants were recruited from a larger study that took place over 2 years and was a 12-week prospective case-control study, with at least 12-week follow-up. It compared the diagnostic yield of PAF, in a population with symptoms of possible AF, using either a continuous automated cardiac event recorder (the R Test 4 Evolution, Novacor; 1-week test period) or a hand-held, battery-driven ECG recorder (Omron HCG-801; Omron Healthcare, United Kingdom). The study was approved by National Research Ethics Service Committee (12/LO1357) and the Royal Surrey County Hospital Research & Development committee. Participants were recruited over 21 months by primary care physicians in the Waverley Health District. Participants gave informed consent and were given a study number to anonymise data. Methods, data collection and storage were performed according to relevant guidelines and regulations in the Research Governance Framework for Health and Social Care (NHS Health Research Authority, 2018) and conformed to updated (March 2018) United Kingdom Policy Frameworks for Health and Social Care Research. All primary data were stored in encrypted and password-protected computers.

### Participant Eligibility Criteria and ECG Collection

Inclusion criteria were: presenting with palpitations or an irregular pulse; age ≥ 40 years; no history of AF; no electrolyte abnormalities; no pacemaker device; no prescribed class Ic or III anti-arrhythmic drugs; no other arrhythmias. Controls had no evidence of PAF during the study period. Cases had PAF diagnosed with either device during the main study, recordings for this sub-study were made prior to initiation of any antiarrhythmic drug. PAF was defined as AF lasting 30-s to 7 days with spontaneous termination. Fifty-seven patients (30 cases; 27 controls) were recruited. The cardiologist (PH) reported on ECG data throughout the study and categorized participants as controls or cases. Table 1 lists demographics, clinical data and current medications.

**TABLE 1:** Demographic and clinical data.

	Controls (*n* = 24)	Cases (*n* = 28)
Age, years	66.3 ± 9.9	70.8 ± 7.4
Male:Female	8M:16F	8M:20F
Systolic BP, mmHg	136 ± 21	143 ± 20
Diastolic BP, mmHg	79 ± 13	76 ± 13
Heart rate, min^–1^	74 ± 10	71 ± 11
BMI, kg.m**^–^**^2^	25.1 ± 4.3	26.8 ± 4.5
CHA_2_DS_2_-VASc score	2.1 ± 1.5	2.3 ± 1.3
Smokers	3	6
Excess alcohol	3	2
Diabetes mellitus	1	0
Ischaemic heart disease.	1	4
Cardiac failure	0	0
Stroke/TIA	0	1
Dyslipidaemia	9	11
Total medications	1.8 ± 1.7	3.3 ± 2.7*
All antihypertensive agents	9	10
β-blockers	0	10*
Warfarin	2	1
Aspirin/clopidogrel	0	10*

Mean values ± SD or numbers within each cohort. CHA_2_DS_2_-VASc score calculated from Lip et al. (2010); BMI, basal metabolic index; TIA, transient ischaemic attack; all antihypertensives exclude β-blockers. *p < 0.05 χ^2^-test.

Participants recorded 28-s ECG periods (strips) with the Omron recorder twice-daily in a rested state whilst sitting, at roughly 12-h intervals, initially over a period of 5 weeks although some provided more. Initial data evaluation from eight control and seven case participants showed at least 30 strips per participant were required, more provided little additional benefit – see Results. Subsequently, participants were asked to provide recordings over 3 weeks (42 strips) – the signal-to-noise ratio was 15–20 dB. From 57 original participants, 52 (28 cases; 24 controls) provided ≥33 strips for analysis. Four were excluded because four participants provided <30 strips and with one participant base-line drift and extraneous electrical noise during recording was present. The cardiologist also confirmed that traces were representative of sinus rhythm, with no evidence of AF or ventricular abnormalities (dysrhythmias, ectopics, or abnormal waveforms).

### Conversion of ECG Recordings to Binary Strings and Analysis

**FIGURE 1:**
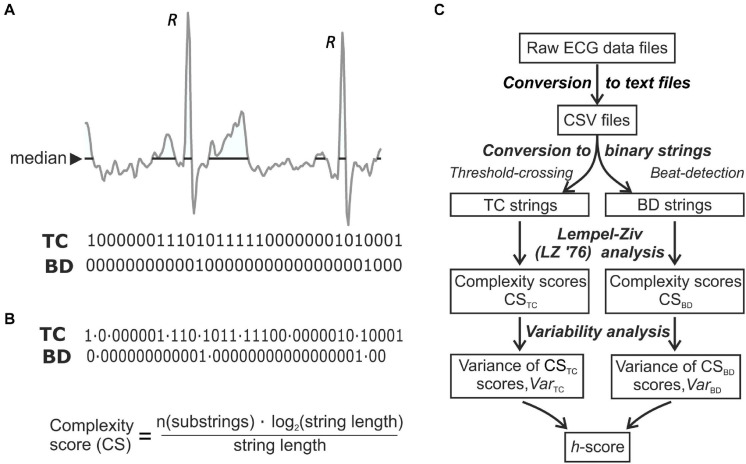
ECG analysis techniques. **(A)** The two methods to convert a digitised ECG recording to a binary string. Threshold Crossing (TC) substitutes **“**1**”** for all values equal to or above a median threshold and sets all other values to **“**0.**”** Beat Detection (BD) sets all values of a binary string to zero except at time-points where the R-wave peak is detected; to *R*-waves are marked on the trace. **(B)** The binary strings were split into a set of unique substrings (LZ’76 complexity analysis); the final complexity score was normalized to the length of the recording. **(C)** Flowchart of ECG processing to obtain a final discriminating *h*-score.

The Omron device is a bipolar, single-channel recorder sampling at 125 Hz with signal bandwidth 0.05–40 Hz. Analyses were enabled by custom-built programs developed in C++ using a Qt framework^1^. The first used documentation provided by Omron, under a non-disclosure agreement, that converted recordings from the proprietary file format to comma-separated-values (*csv*) text files. Files retained only anonymised information essential for further data processing. The second analyzed *csv* files by converting floating-point ECG recordings into binary strings, to calculate Lempel–Ziv complexity scores (CS) using two algorithms (Figures 1A,B).

The TC method used a threshold-crossing algorithm replacing all values above a threshold by “1” and setting the rest to “0.” The median value of each strip was used as a threshold due to its insensitivity to outliers. The BD (beat detection) method used a QRS complex detection algorithm that assigned a unitary value for each R peak. The first derivative (d*V*/d*t*) of the ECG voltage was generated and smoothed, by a process of convolution with a digital Savitsky-Golay filter (Savitzky and Golay, 1964) with a window size of “5,” that increased precision without distorting the signal (Nishida et al., 2017; Sadeghi and Behnia, 2018). A sliding window corresponding to 6 s of strip duration was moved along the signal and the maximum value of d*V*/d*t* (d*V*/d*t*_max_) was found within the window. Then the first sample within the window which satisfied two criteria was taken as the R-peak time and assigned a value of “1,” with all other points a value of “0.” The criteria were: i) d*V*/d*t* > 0.7^∗^d*V*/d*t*_max_ in the window and ii) d*V*/d*t* is greater than both preceding and succeeding values. The window was then advanced and the process repeated. This simple technique was acceptable in recordings lacking artifacts and rhythm irregularities and the algorithm is at heart rates below 100 min^–1^ (Alexeenko et al., 2019). In this study heart rates for all participants were <100 min^–1^ [controls; 74 ± 2 (SEM, range 60–91) min^–1^, *n* = 24: cases; 73 ± 1 (SEM, range 59–88) min^–1^, *n* = 28].

### Lempel-Ziv Complexity and the Final Outcome Measure, the h-Score

Lempel-Ziv (LZ’76) complexity is a non-linear signal analysis method to estimate sequence complexity (CS; Lempel and Ziv, 1976) by identifying the number of different sub-sequences and their recurrence rate (Radhakrishnan and Gangadhar, 1998). The ECG time series, *x*(i) was converted to a discrete, binary, sequence, *P* = *s*(1),*s*(2) by comparing *x*(*i*) with a threshold *T*_*d*_ with *s*(*i*):

(1) $$s\left(i\right)=\{\frac{0\,i\,f\,x\,\left(i\right)<T_{d}}{1\,i\,f\,x\,\left(\text{i}\right)\geq T_{d}}$$

LZ’76 complexity was estimated by scanning *P* from left to right and increasing a complexity counter *c*(*n*) with every new sub-sequence (Lempel and Ziv, 1976; Kaspar and Schuster, 1987). To achieve independence of *c*(*n*) from sequence length (*n*), the number of unique sub-sequences was normalized to the *n*/log_2_(*n*) = *b*(*n*) value (Hand, 1981; Figure 1B), i.e., *CS*(*n*) = *c*(*n*)/*b*(*n*). Thus, *CS*_TC_ and *CS*_BD_ scores were generated for each strip. Next variability (*varCS*_TC_ or *varCS*_BD_) scores for each patient were calculated as CS variability discriminated better between the two cohorts. Thus *varCS*_TC_ = Σ(*iCS*_TC_–mean*CS*_TC_)^2^, where *iCS*_TC_ is an individual CS (same for *CS*_BD_). The final discriminant measure, the *h*-score was calculated and reflects the independent variability of *CS*_TC_ and *CS*_BD_ scores for each participant during sinus rhythm. With a constant, *k*

(2) $$h-s\,c\,o\,r\,e=\sqrt{v\,a\,r\,C\,S_{T\,C}^{2}+k.v\,a\,r\,C\,S_{B\,D}^{2}}$$

A flow chart of the analysis is shown in Figure 1C, see Results for calculation of *k*.

The enclosed Supplement contains the source code for the LZ’76 complexity estimator used in this analysis. The Supplement also includes data sets used for validation as well as the expected program outputs.

### Statistical Analysis

Not all summary data sets for *CS*_TC_/*CS*_BD_, their derived variability scores or the final *h*-score were normally distributed (Shapiro-Wilks tests) and so these data are quoted as medians with 25 and 75% interquartiles. Differences between controls and cases cohorts were calculated with Mann–Whitney *U*-tests: the null hypothesis was rejected at *p* < 0.05. Mean values of *CS*_TC_/*CS*_BD_ for each participant were used to calculate varCS_TC_ or varCS_BD_ scores. Intra-subject analyses showed CS_TC_ scores were normally distributed, except for one in each cohort with excess kurtosis (*k*) > 1. For CS_BD_ scores, *k* > 1 with two controls, 12 cases; skewness (s) > | 1| for two cases participants. Categorical data sets were compared with a χ^2^-analysis. Receiver-Operating Characteristic (ROC) empirical curves described test characteristics, with area-under-the-curve (AUC) as a summary statistic^2^. Significance between different AUCs was also tested^3^. The operating point of the final AUC for the *h*-score was the point where a 45° line is tangent to the ROC curve. A Spearman rank-order correlation coefficient, *r*_s_, was calculated to test association between two variables. Data summaries and statistical analyses were performed using Excel or Vassar Stats.

## Results

### Participant Characteristics

Participants were divided into cases or controls who did or did not show eventual evidence of paroxysmal atrial fibrillation, but during recording were in sinus rhythm. The two cohorts were statistically similar for all demographic and relevant clinical data (Table 1). Pharmacotherapy showed that total medications in the cases cohort were greater, in particular for β-blockers (10/28 vs 0/24) and aspirin/clopidogrel (9/28 vs 0/24). Initial sub-analyses revealed no differences in ECG complexity metrics in the cases cohort between those taking β-blockers or aspirin/clopidogrel and those who did not, thus all data in this cohort were combined.

### Complexity Score Values

**FIGURE 2:**
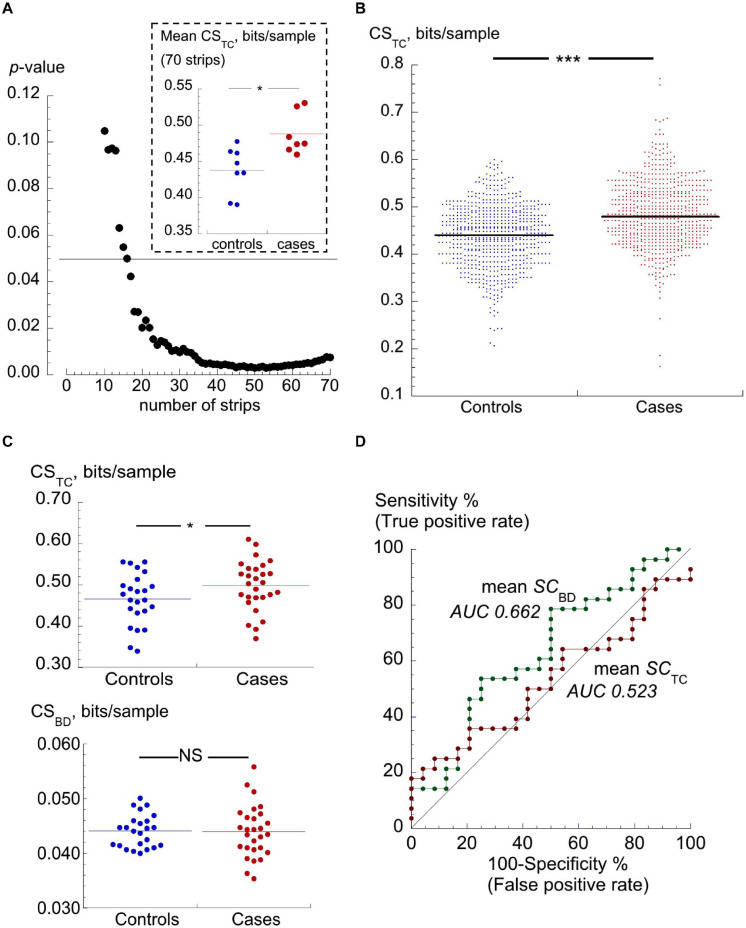
TC and BD complexity scores. **(A)** *p*-values of differences between mean CS_TC_ values between a group of controls (*n* = 8) and cases (*n* = 7) participants who each offered >75 ECG strips. *p*-values calculated after successive final strips from the first 70 strips were removed. The horizontal line shows the value for *p* = 0.05. The inset shows mean values of the CS_TC_ values for these subsets of participants, *p < 0.05. **(B)** Dot plots of CS_TC_ scores of individual ECG traces (strips) from 24 controls and 28 PAF cases. Horizontal lines mark the median values of scores, ***p < 0.001. **(C)** Dot plots of median CS_TC_ (upper) and median CS_BD_ (lower) values for individual participants. The horizontal lines through the data points show median values of each set; *p < 0.05. **(D)** ROC curves using CS_TC_ and CS_BD_ for discrimination between controls and cases cohorts; AUC values shown by each curve.

Digitised ECG strips were downloaded and the two Lempel-Ziv CSs (CS_TC_ and CS_BD_, see section “Materials and Methods”) calculated. An initial evaluation of the number of strips required from each participant was carried out with 15 participants (controls *n* = 8; cases *n* = 7) who all provided more than 75 strips. Average CS_TC_ was calculated for each participant with the final strip successively removed until only the first 10 were used. The *p*-value for the difference between the two cohorts showed that discrimination was increasingly lost with fewer than 30 strips per participant (Figure 2A). The inset shows the mean of CS_TC_ values from the two cohorts when the first 35 strips from each patent were used.

The median CS_TC_ score off all strips from 24 control and 28 cases participants was significantly greater in ECG strips from cases vs controls [0.488 (0.434, 0.548) bits/sec, *n* = 1571 ECG strips vs 0.464 (0.410, 0.515) bits/sec, *n* = 1392; *p* < 0.001: Figure 2B]. Median CS_BD_ scores in both cohorts were the same although the two sets were significantly different due a greater range of values in the cases cohort [0.0437 (0.0404, 0.0471) bits/sec, *n* = 1571 vs 0.0437 (0.0404, 0.0471) bits/sec, *n* = 1392; *p* = 0.039]. However, neither score alone provided a useful discriminator due to the considerable overlap of values between the two cohorts, as exemplified by the CS_TC_ data sets in Figure 2B.

Mean values of CS_TC_ and CS_BD_ for each participant were calculated: mean-CS_TC_ values remained significantly (*p* = 0.039) different between cases and controls (Table 2) but mean-CS_BD_ scores were not significantly different (*p* = 0.92); Figure 2C. The usefulness of mean CS_TC_ and CS_BD_ scores for identifying future PAF subjects was assessed using a ROC curve analysis (Figure 2D); neither was a good discriminator between cases and controls with respective area-under-the-curve (AUC) values of 0.662 and 0.523 (Table 2).

**TABLE 2:** Complexity scores (CS) for threshold crossing (TC) and beat detection (BD), their derivative variabilities, *var*CS_TC_ and *var*CS_BD_, as well as final *h*-scores for data from controls (*n* = 24) and cases (*n* = 28) cohorts.

	Controls	Cases	AUC (C.I.)
CS_TC_	0.470 (0.436, 0.502)	0.504 (0.469, 0.542)*	0.662 (0.511–0.813)
CS_BD_	0.0443 (0.0414, 0.0459)	0.0439 (0.0409, 0.0472)	0.490 (0.329–0.651)
*var*CS_TC_	3.23 (2.43, 3.64)⋅10^–3^	4.21 (2.84, 5.38)⋅10^–3^**	0.740 (0.604–0.876)
*var*CS_BD_	2.05 (1.75, 2.60)⋅10^–5^	3.15 (2.43, 3.94)**	0.798 (0.677–0.919)
*h*-score	3.87 (3.39, 4.37)⋅10^–3^	5.93 (4.85, 7.06)⋅10^–3^***	0.919 (0.844–0.994)

Data are medians (25,75% interquartiles); *p < 0.05, **p < 0.01, and ***p < 0.001. The final column shows AUC estimations from ROC curve analysis and 95% confidence intervals (C.I.) of differentiation between the two cohorts.

### Variability of CS Scores

**FIGURE 3:**
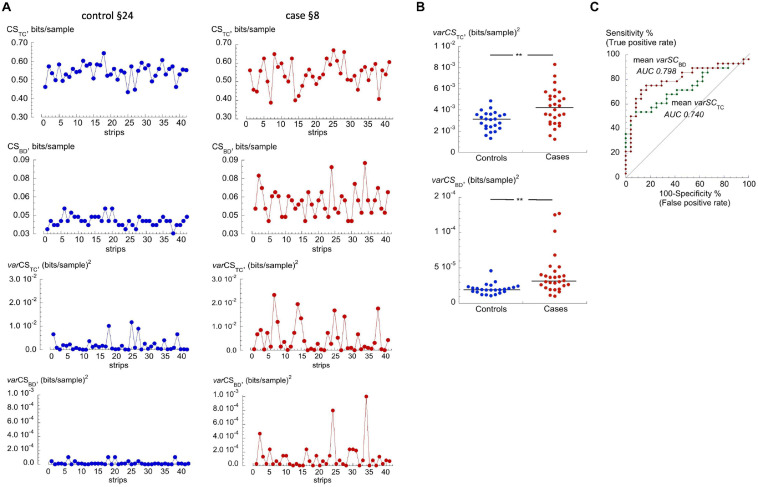
Variability of CS_TC_ and CS_BD_. **(A)** Data from for a ‘control (§24)’ and a ‘cases (§8)’ participants. Shown (top to bottom) are CS_TC_, CS_BD_, *var*CS_TC_ and *var*CS_BD_ values over the recording period. **(B)** Values of median *var*CS_TC_ and *var*CS_BD_ for each participant in the control and cases cohorts; **p < 0.005. **(C)** ROC curves for *var*CS_TC_ and *var*CS_BD_ for discrimination between controls and cases cohorts. AUC values shown by each curve.

Variability of individual CS_TC_ and CS_BD_ values (*var*CS_TC_ or *var*CS_BD_; units, (bits/sample)^2^ for a participant were greater in the cases cohort compared to those in the control cohort. Generally, those in the control cohort had fewer outliers and more uniform complexity values than those in the cases cohort. Figure 3A shows examples of CS_TC_, CS_BD_ and respective *var*CS_TC_ or *var*CS_BD_ values from a control participant (§24), who showed little variability, and a cases participant (§8) with more variability. Median *var*CS_TC_ or *var*CS_BD_ values were both significantly greater for the cases cohort (*p* = 0.00147, *p* = 0.00148, respectively, Figure 3B and Table 2). ROC curve analysis of *var*CS_TC_ and *var*CS_BD_ performance as binary classifiers showed increased AUC values over the base CS scores (Figure 3C). The *var*CS_TC_ AUC = 0.740, but was not statistically different from the CS_TC_ value (*p* = 0.22). However, the *var*CS_BD_ AUC (= 0.798) was significantly (*p* = 0.001) improved.

### Calculation of the Final Discriminant Score

**FIGURE 4:**
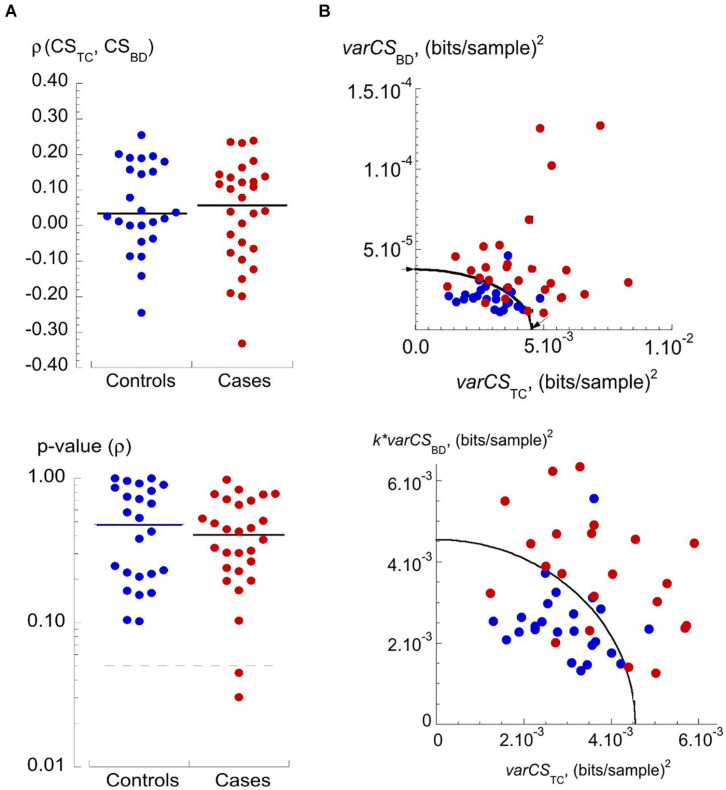
Independence of *var*CS_TC_ and *var*CS_BD_ and their values for controls and cases. **(A)** Dot plots of Spearman rank correlation coefficient, *ρ*, (upper) and calculated *p*-values (lower) of *var*_TC_-*var*_BD_ relationships in the controls and cases cohorts. Lower plot: dotted line represents the *p*-value of 0.05; note the logarithmic ordinate scale. **(B)** Upper: plot of *var*CS_BD_ vs *var*CS_TC_ values for controls (blue circles) and cases (red circles). The curve is an ellipse that optimally separates data from controls and cases cohorts, arrows mark the intercepts with axes, used to estimate a scaling factor for the *var*CS_BD_ data – see text for details. Lower plot: transformed data where the *var*CS_BD_ data are multiplied by a constant, *k*, to allow a circle function to optimally separate data from controls and cases cohorts – see text for details.

A key observation was that *var*CS_TC_ and *var*CS_BD_ values were uncorrelated for data from a particular participant. Figure 4A plots Spearman correlation coefficients (ρ) and corresponding *p*-values for *var*CS_TC_ and *var*CS_BD_ pairs from individual participants. There was no significant association between these two variance scores for any individual, except for two (in the cases cohort) where significance was just achieved. Overall, the two variance scores could be used as independent variables. The mean values of *var*CS_BD_ vs *var*CS_TC_ (Figure 4B, top) showed a clustering of data from the control cohort in the lower left-hand quadrant. Also shown is an ellipse function that optimally separates data points from the two cohorts and with intersections on the two axes at *var*CS_TC_ = 4.546.10^–3^ and *var*CS_BD_ = 3.77.10^–5^ (Figure 4B, top – arrowed). To weight equally the two variance measures, *var*CS_BD_ values were normalized by multiplying by *k* = 120.6; the ratio of the two intercepts. Figure 4B (lower) shows the data transformation now with a circle fit of radius 0.00455 bits/sample^2^; note that only the sub-set of data points near the circle boundary is shown.

**FIGURE 5:**
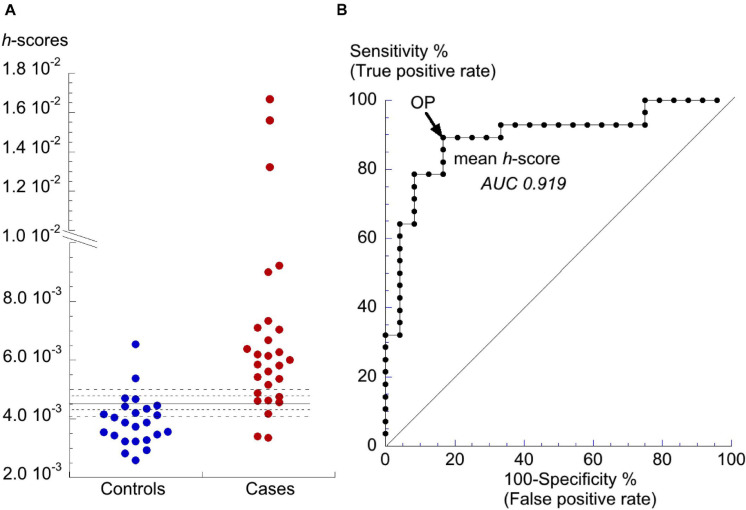
Distribution of h-scores and final discriminant analysis. **(A)** Dot-plot of *h*-scores for controls and cases. The horizontal line is the value for *h* that optimally separates data from the controls and cases cohorts. The dotted lines represent ±5 and ±10% changes to the *h*-score. **(B)** ROC curve for *h*-scores to discriminate between controls and cases cohorts. AUC value shown by the curve. The operating point (OP) is shown by the arrow.

Finally, to reduce the dimensionality of the data a coordinate transform (Hand, 1981) was applied to produce a single *h*-score which quantified the compound variability of CS_TC_ and CS_BD_ for a participant as the length of the vector from the origin to a particular datum point. Values of *h*-scores are shown in Figure 5A with the decision threshold for the *h*-score = 4.5⋅10^–3^ shown by the solid horizontal line. Controls and cases were separated with 89% sensitivity (true positive rate) and 83% specificity (true negative rate). ROC curve analysis demonstrated the further superiority of the *h*-score (*p* = 0.11 vs *var*CS_TC_ and *p* = 0.049 vs *var*CS_BD_) as the discriminant with an AUC = 0.919 (C.I. 0.844–0.994); Figure 5B and Table 2.

Figures 4B, 5A show that several of the patient *h*-scores lie close to the discriminant boundary (*h*-score = 4.5⋅10^–3^) and a small variation of this value could have important consequences of sensitivity and selectivity estimations. The horizontal dotted lines of Figure 4C show values of the *h*-score varied by ±5 and ±10%.

A decrease of the *h*-score would decrease the number of false negatives but increase the number of false positives. For a 5 and 10% decrease, sensitivity was either unchanged or increased to 93%, respectively, but specificity was reduced to 71 or 58%, respectively. For a 5 and 10% increase, sensitivity fell to 79 or 71%, respectively, but with increased specificity to 92% in both cases.

The CHA_2_DS_2_-VASc score is used to estimate stroke risk in patients with non-rheumatic AF and may offer a further independent score to predict the occurrence of PAF. Any association between the CHA_2_DS_2_-VASc score and the *h*-score was tested by calculation of a Spearman rank-order correlation coefficient, ρ: there was no statistical association for the whole data set (ρ = −0.0443, *p* = 0.758, *n* = 52). Thus, combination of the *h*-score with the CHA_2_DS_2_-VASc score would not provide further discrimination between the two cohorts.

## Discussion

This study shows that analysis of ECG entropy, using LZ’76 complexity, has potential for diagnosing PAF from sinus rhythm ECGs. Analysis of at least 30 half-minute strips per patient, acquired using an inexpensive handheld ECG monitor in patients whilst in sinus rhythm, produced a final score (the *h*-score) based on individual variability of two ECG complexity measures, *CS*_TC_ and *CS*_BD_. Mean *CS*_TC_ scores were statistically different in cases compared to control, whereas mean *CS*_BD_ scores were not, but alone they provided poor discrimination between the two cohorts. Generation of the *h*-score depended on two key observations of this pair of CSs. First, variability of both *CS*_TC_ and *CS*_BD_ (*varCS*_TC_ and *varCS*_BD_) on a day-to-day basis were greater in the cases cohort compared to controls (Figure 3 and Table 2). Second, *varCS*_TC_ and *varCS*_BD_ were independent measures of complexity which enabled generation of a final *h*-score that provided excellent discrimination between the two cohorts with 83% specificity and 89% sensitivity. The *h*-score is an absolute value derived from this pilot study of a relatively small cohort of 52 patients, so that a larger study will generate a value with greater confidence. The observation of greater variability of ECG complexity in patients at risk of PAF implies that their atria show subtle electrophysiological changes that, without immediate gross pathophysiological consequence, provide a substrate or trigger for a period of atrial fibrillation.

Participant compliance was good, 93% provided sufficient numbers of recordings and only one provided data that could not be analysed. The requirement to produce short recordings in a restful home-setting, using a hand-held device will have contributed to this high rate of participation. Because, the key component of the analysis is measurement of CS variability on a day-to-day basis to generate a discriminant score, fewer but longer individual recordings may not be useful. An alternative refinement may be to determine if multiple-lead ECGs or alternative complexity estimates are better, but this remains to be explored. Other strategies might be derived to process higher quality recordings, for example, using more complex ECG parsing techniques but simpler analysis methods (Alexeenko et al., 2020). The method also required a cardiologist to scrutinize ECG traces before analysis to exclude abnormalities, and although they are relatively uncommon in a general population (Sirichand et al., 2017) a fully automated process would need some preliminary screening process (see Limitations, below). Finally, combination with other approaches, including biomarkers such as brain natriuretic peptide (Rodríguez-Yáñez et al., 2013) or AF-related stroke-risk scores might also provide further discrimination. However, combination of the *h*-score with CHA_2_DS_2_-VASc scores provided no improvement of selectivity or sensitivity.

The relative simplicity of this predictive method makes it suitable for population screening of at-risk groups or those who cannot co-operate easily with clinical tests. The method may also be applied to analyze previously-collected data, e.g., to investigate links between subclinical AF and cryptogenic stroke (Healey et al., 2012) or development of dementia, when early screening would be especially useful (Cuadrado-Godia et al., 2020).

### Methods of Atrial Fibrillation Detection

This method of AF prediction, using simple and unambiguous ECG parsing algorithms coupled to second moment analysis of short and relatively low-quality ECGs recorded by hand-held devices may be compared to other methods that measure existing AF and potentially predict its occurrence. Measurement of existing AF is continuously improving and achieves similar sensitivity and selectivity to that recorded here, even with hand-held devices (Svennberg et al., 2015; Marinucci et al., 2020); however, their use to record paroxysmal AF is limited. Alternatively, analysis of risk factors that combine demographic features, simple clinical tests and plasma biomarkers, through generation of machine-learning models are increasingly sophisticated (Ambale-Venkatesh et al., 2017; Hill et al., 2019). They have the advantage of yielding pathological insight but require sophisticated resources and thus far are less sensitive and selective.

More precise electrophysiological approaches are also being developed. Machine-learning methods for PAF detection use retrospective analysis of freely available clinical ECG recordings or clinical databases. These are often based on detection and classification of atrial premature beats and other ECG abnormalities (Thong et al., 2004), or from interval analysis of atrial or ventricular depolarisations (Ghodrati et al., 2008; Mohebbi and Ghassemian, 2012; Xin and Zhao, 2017; Aronis et al., 2018), with specificity and sensitivity ranging between 71–93% and 85–96%, respectively, but requiring recording periods up to 30 min. However, convolutional neural networks achieve accuracy of detection in the range 75–95% using shorter recording periods of detection (Hsieh et al., 2020; Nurmaini et al., 2020). Finally, machine-learning predictive methods using sinus rhythm recordings are also being developed with sensitivity and selectivity around 83% (Attia et al., 2019).

### A Health Economics Perspective

An estimate of net monitoring costs for AF over 1 week after an ischaemic stroke, has been estimated to be about $530,000 at today’s costs (Kamel et al., 2010). Outlay for 1,000 re-useable hand-held monitors of about $125,000, plus employment of a biometrics analyst represents a large saving to health-care systems to identify vulnerable patients at risk of subsequent strokes from PAF.

### Limitations

(i): Due to the nature of PAF and with intermittent monitoring, some individuals assigned as controls may have undetected PAF. All cases had at least one PAF episode during the study period, but some controls may have experienced PAF at greater intervals. (ii): Recorded co-morbidities were similar in both groups, but more cases than controls took β-blockers and/or aspirin or clopidogrel. (iii): Participant compliance; of the original 57 participants two each of controls and cases supplied < 30 strips for analysis and with one control artifacts precluded analysis. (iv): External electrical noise, e.g., from electromyographic activity of the participant’s hand, could add to the ECG signal and if excessive alter the TC complexity score (CS_TC_). We added noise to 15 ECG trace segments from 10 random participants (five each of controls and cases) and recalculated CS_TC_. It showed that when noise exceeded 181 μV SD (equivalent to a signal-to-noise ratio of 15.9 dB) mean complexity scores were altered by more than 2.5%. Therefore, the analysis will be useful for signals with a signal-to-noise ratio > 15.9 dB. (v): The nature of this pilot study precluded recruitment of additional patients comprising a validation cohort, so this remains a proof-of-principle study. A larger case-control study is required to validate the predictive power of the *h*-score. (vi): The BD algorithm used in this study was sufficiently robust to detect specifically R-waves in ECG traces showing sinus rhythm. However, ventricular dysrhythmias or waveform abnormalities, such as bigeminy or T-wave alternans, may be confounders that contribute false positives. In this proof-of-principle study, clinical evaluation would have excluded such traces; however, this would be unsuitable for a fully automated process. We envisage the next phase is to incorporate a preliminary ECG parsing step, for example with a Pan-Tompkins parser (Pan and Tompkins, 1985), supplemented with an autocorrelation analysis, to identify traces with these potential confounders.

## Conclusion

We describe a link between increased variability of ECG complexity in sinus rhythm recordings and PAF incidence and propose a novel score to quantify PAF risk. We envisage this score would enable low-cost screening for PAF based on short periods of ECG recording in a primary care setting or built into hand-held devices. We anticipate such screening would improve detection of PAF relative to currently available techniques (Choe et al., 2015). This may contribute to a reduction of AF-related mortality that, unlike for heart failure, continues to rise, at least in Europe and United States (Vasan et al., 2019).

## Data Availability Statement

The original contributions presented in the study are included in the article/Supplementary Material, further inquiries can be directed to the corresponding author/s.

## Ethics Statement

The studies involving human participants were reviewed and approved by the study was approved by National Research Ethics Service Committee (12/LO1357) and the Royal Surrey County Hospital Research Development committee. Participants were recruited over 21 months by primary care physicians in the Waverley Health District. Participants gave informed consent and were given a study number to anonymise data. Methods, data collection and storage were performed according to relevant guidelines and regulations in the Research Governance Framework for Health and Social Care (NHS Health Research Authority, 2018) and conformed to updated (March 2018) United Kingdom Policy Frameworks for Health and Social Care Research. All primary data were stored in encrypted and password-protected computers. The patients/participants provided their written informed consent to participate in this study.

## Author Contributions

CF, VA, and RJ: devised the study. CF, VA, JF, DA, and RJ: experimental planning. VA, PH, and JF: contributed to the data. RJ and CF: raised funding. CF, VA, and RJ: drafted the manuscript. All authors edited and approved the final manuscript.

## Conflict of Interest

The authors declare that the research was conducted in the absence of any commercial or financial relationships that could be construed as a potential conflict of interest.

## Footnotes

1 http://www.qt.io

2 http://www.rad.jhmi.edu/jeng/javarad/roc/JROCFITi.html

3 http://vassarstats.net/roc_comp.html

## References

[B1] Aarabi A. He B. (2017). Seizure prediction in patients with focal hippocampal epilepsy. *Clin. Neurophysiol.* 128 1299–1307. 10.1016/j.clinph.2017.04.026 28554147 PMC5513720

[B2] Abásalo D. Simons S. Morgado da Silva R. Tonon G. Vyazovskiy V. V. (2015). Lempel-Ziv complexity of cortical activity during sleep and waking in rats. *J. Neurophysiol.* 113 2742–2752. 10.1152/jn.00575.2014 25717159 PMC4416627

[B3] Alexeenko V. Fraser J. A. Bowen M. Huang C. L. H. Marr C. M. Jeevaratnam K. (2020). The complexity of clinically-normal sinus-rhythm ECGs is decreased in equine athletes with a diagnosis of paroxysmal atrial fibrillation. *Sci. Rep.* 10:6822. 10.1038/s41598-020-63343-7 32321950 PMC7176685

[B4] Alexeenko V. Fraser J. A. Dolgoborodov A. Bowen M. Huang C. L. Marr C. M. (2019). The application of Lempel-Ziv and Titchener complexity analysis for equine telemetric electrocardiographic recordings. *Sci. Rep.* 9:2619. 10.1038/s41598-019-38935-7 30796330 PMC6385502

[B5] Ambale-Venkatesh B. Yang X. Wu C. O. Liu K. Hundley W. G. McClelland R. (2017). Cardiovascular event prediction by machine learning: the multi-ethnic study of atherosclerosis. *Circ. Res.* 121 1092–1101. 28794054 10.1161/CIRCRESAHA.117.311312 PMC5640485

[B6] Amigo J. M. Szczepanski J. Wajnryb E. Sanchez-Vives M. V. (2004). Estimating the entropy rate of spike trains via Lempel-Ziv complexity. *Neural Comput.* 16 717–736. 10.1162/089976604322860677 15025827

[B7] Andrade J. Khairy P. Dobrev D. Nattel S. (2014). The clinical profile and pathophysiology of atrial fibrillation: relationships among clinical features, epidemiology, and mechanisms. *Circ. Res.* 114 1453–1468. 10.1161/CIRCRESAHA.114.303211 24763464

[B8] Aronis K. N. Berger R. D. Calkins H. Chrispin J. Marine J. E. Spragg D. D. (2018). Is human atrial fibrillation stochastic or deterministic? Insights from missing ordinal patterns and causal entropy-complexity plane analysis. *Chaos* 28:063130. 10.1063/1.5023588 PMC6026026 29960392

[B9] Attia Z. I. Noseworthy P. A. Lopez-Jimenez F. Asirvatham S. J. Deshmukh A. J. Gersh B. J. (2019). An artificial intelligence-enabled ECG algorithm for the identification of patients with atrial fibrillation during sinus rhythm: a retrospective analysis of outcome prediction. *Lancet* 394 861–867. 10.1016/S0140-6736(19)31721-0 31378392

[B10] Banerjee A. Taillandier S. Olesen J. B. Lane D. A. Lallemand B. Lip G. Y. (2013). Pattern of atrial fibrillation and risk of outcomes: the Loire Valley Atrial Fibrillation Project. *Int. J. Cardiol.* 167 2682–2687. 10.1016/j.ijcard.2012.06.118 22795403

[B11] Choe W. C. Passman R. S. Brachmann J. Morillo C. A. Sanna T. Bernstein R. A. (2015). A comparison of atrial fibrillation monitoring strategies after cryptogenic stroke. *Am. J. Cardiol.* 116 890–893. 10.1016/j.amjcard.2015.06.012 26183793

[B12] Cuadrado-Godia E. Benito B. Ois A. Vallès E. Rodríguez-Campello A. Giralt-Steinhauer E. (2020). Ultra-early continuous cardiac monitoring improves atrial fibrillation detection and prognosis of patients with cryptogenic stroke. *Eur. J. Neurol.* 27 244–250. 10.1111/ene.14061 31424609

[B13] Dulli D. A. Stanko H. Levine R. L. (2003). Atrial fibrillation is associated with severe acute ischemic stroke. *Neuroepidemiology* 22 118–123. 10.1159/000068743 12629277

[B14] Ganesan A. N. Chew D. P. Hartshorne T. Selvanayagam J. B. Aylward P. E. Sanders P. (2016). The impact of atrial fibrillation type on the risk of thrombo-embolism, mortality, and bleeding: a systematic review and meta-analysis. *Eur. Heart J.* 37 1591–1602. 10.1093/eurheartj/ehw007 26888184

[B15] Ghodrati A. Murray B. Marinello S. (2008). RR interval analysis for detection of atrial fibrillation in ECG monitors. *Conf. Proc. IEEE Eng. Med. Biol. Soc.* 2008 601–604. 10.1109/IEMBS.2008.4649224 19162727

[B16] Glass L. (2009). Introduction to controversial topics in nonlinear science: is the normal heart rate chaotic? *Chaos* 19:028501. 10.1063/1.3156832 19566276

[B17] Goldberger A. (1991). Is the normal heartbeat chaotic or homeostatic? *Physiology* 6 87–91. 10.1152/physiologyonline.1991.6.2.87 11537649

[B18] Han T. S. Fry C. H. Fluck D. Affley B. Gulli G. Barrett C. (2018). Anticoagulation therapy in patients with stroke and atrial fibrillation: a registry-based study of acute stroke care in Surrey, UK. *BMJ Open* 8:e022558. 10.1136/bmjopen-2018-022558 29997144 PMC6089275

[B19] Hand D. J. (1981). *Discrimination and Classification. Wiley Series in Probability in Mathematical Statistics.* Chichester: Wiley.

[B20] Hart R. G. Pearce L. A. Aguilar M. I. (2007). Meta-analysis: antithrombotic therapy to prevent stroke in patients who have nonvalvular atrial fibrillation. *Ann. Intern. Med.* 146 857–867. 10.7326/0003-4819-146-12-200706190-00007 17577005

[B21] Hart R. G. Sharma M. Mundl H. Kasner S. E. Bangdiwala S. I. Berkowitz S. D. (2018). Rivaroxaban for stroke prevention after embolic stroke of undetermined source. *N. Engl. J. Med.* 378 2191–2201. 10.1056/NEJMoa1802686 29766772

[B22] Healey J. S. Connolly S. J. Gold M. R. Israel C. W. Van Gelder I. C. Capucci A. (2012). Subclinical atrial fibrillation and the risk of stroke. *N. Engl. J. Med.* 366 120–129. 22236222 10.1056/NEJMoa1105575

[B23] Hill B. R. Ayoubkhani D. McEwan P. Sugrue D. M. Farooqui U. Lister S. (2019). Predicting atrial fibrillation in primary care using machine learning. *PLoS One* 14:e0224582. 10.1371/journal.pone.0224582 31675367 PMC6824570

[B24] Hornero R. Abasolo D. Escudero J. Gomez C. (2009). Nonlinear analysis of electro-encephalogram and magnetoencephalogram recordings in patients with Alzheimer’s disease. *Philos. Trans. A Math. Phys. Eng. Sci.* 367 317–336. 10.1098/rsta.2008.0197 18940776

[B25] Hsieh C. H. Li Y. S. Hwang B. J. Hsiao C. H. (2020). Detection of atrial fibrillation using 1D convolutional neural network. *Sensors* 20:2136. 10.3390/s20072136 32290113 PMC7180882

[B26] Jorfida M. Antolini M. Cerrato E. Caprioli M. G. Castagno D. Garrone P. (2016). Cryptogenic ischemic stroke and prevalence of asymptomatic atrial fibrillation: a prospective study. *J. Cardiovasc. Med.* 17 863–869. 10.2459/JCM.0000000000000181 25379716

[B27] Kamel H. Hegde M. Johnson D. R. Gage B. F. Johnston S. C. (2010). Cost-effectiveness of outpatient cardiac monitoring to detect atrial fibrillation after ischemic stroke. *Stroke* 41 1514–1520. 10.1161/STROKEAHA.110.582437 20508188

[B28] Kaspar F. Schuster H. G. (1987). Easily calculable measure for the complexity of spatiotemporal patterns. *Phys. Rev. A Gen. Phys.* 36 842–848. 10.1103/physreva.36.842 9898930

[B29] Kirchhof P. Benussi S. Kotecha D. Ahlsson A. Atar D. Casadei B. (2016). ESC guidelines for the management of atrial fibrillation developed in collaboration with EACTS. *Europace* 18 1609–1678. 10.1093/eurheartj/ehw210 27567465

[B30] Lempel A. Ziv J. (1976). Complexity of finite sequences. *IEEE Trans. Inf. Theory* 22 75–81.

[B31] Lip G. Y. Frison L. Halperin J. L. Lane D. A. (2010). Identifying patients at high risk for stroke despite anticoagulation: a comparison of contemporary stroke risk stratification schemes in an anticoagulated atrial fibrillation cohort. *Stroke* 41 2731–2738. 20966417 10.1161/STROKEAHA.110.590257

[B32] Maheshwari A. Norby F. L. Roetker N. S. Soliman E. Z. Koene R. J. Rooney M. R. (2019). Refining prediction of atrial fibrillation-related stroke using the P_2_-CHA_2_DS_2_-VASc score. *Circulation* 139 180–191. 10.1161/CIRCULATIONAHA.118.035411 30586710 PMC6481672

[B33] Marinucci D. Sbrollini A. Marcantoni I. Morettini M. Swenne C. A. Burratini L. (2020). Artificial neural network for atrial fibrillation identification in portable devices. *Sensors* 20:3570. 10.3390/s20123570 32599796 PMC7348709

[B34] Mohebbi M. Ghassemian H. (2012). Prediction of paroxysmal atrial fibrillation based on non-linear analysis and spectrum and bispectrum features of the heart rate variability signal. *Comput. Methods Programs Biomed.* 105 40–49. 10.1016/j.cmpb.2010.07.011 20732724

[B35] Nattel S. Harada M. (2014). Atrial remodeling and atrial fibrillation: recent advances and translational perspectives. *J. Am. Coll. Cardiol.* 63 2335–2345.24. 10.1016/j.jacc.2014.02.555 24613319

[B36] NHS Health Research Authority (2018). *Research Summaries.* Available online at: https://www.hra.nhs.uk

[B37] Nishida E. N. Dutra O. O. Ferreira L. H. C. Golletta G. D. (2017). “Application of Savitzky-Golay digital differentiator for QRS complex detection in an electrocardiographic monitoring system,” in *Proceedings of the 2017 IEEE International Symposium on Medical Measurements and Applications (MeMeA)*, Rochester, MN, 233–238. 10.1109/MeMeA.2017.7985881

[B38] Nurmaini S. Tondas A. E. Darmawahyuni A. Rachmatullah M. N. Partan R. U. Firdaus F. (2020). Robust detection of atrial fibrillation from short-term electrocardiogram using convolutional neural networks. *Fut. Gen. Comput. Syst.* 113 304–317.

[B39] Page R. L. Wilkinson W. E. Clair W. K. McCarthy E. A. Pritchett E. L. (1994). Asymptomatic arrhythmias in patients with symptomatic paroxysmal atrial fibrillation and paroxysmal supraventricular tachycardia. *Circulation* 89 224–227. 10.1161/01.cir.89.1.224 8281651

[B40] Pan J. Tompkins W. (1985). A real-time QRS detection algorithm. *IEEE Trans. Biomed. Eng.* 32 230–236. 3997178 10.1109/TBME.1985.325532

[B41] Peng Z. Genewein T. Braun D. A. (2014). Assessing randomness and complexity in human motion trajectories through analysis of symbolic sequences. *Front. Hum. Neurosci.* 8:168. 10.3389/fnhum.2014.00168 24744716 PMC3978291

[B42] Qu Z. (2011). Chaos in the genesis and maintenance of cardiac arrhythmias. *Prog. Biophys. Mol. Biol.* 105 247–257. 10.1016/j.pbiomolbio.2010.11.001 21078337 PMC3047604

[B43] Radhakrishnan N. Gangadhar B. N. (1998). Estimating regularity in epileptic seizure time-series data: a complexity-measure approach. *IEEE Eng. Med. Biol. Mag.* 17 189–194. 10.1109/51.677174 9604706

[B44] Rodríguez-Yáñez M. Arias-Rivas S. Santamaría-Cadavid M. Sobrino T. Castillo J. Blanco M. (2013). High pro-BNP levels predict the occurrence of atrial fibrillation after cryptogenic stroke. *Neurology* 81 444–447. 10.1212/WNL.0b013e31829d8773 23803318

[B45] Sadeghi M. Behnia F. (2018). Optimum window length of Savitzky-Golay filters with arbitrary order. *arXiv* [Preprint]. arXiv:1808.10489

[B46] Savitzky A. Golay M. J. (1964). Smoothing and differentiation of data by simplified least squares procedures. *Anal. Chem.* 36 1627–1639. 10.1021/ac60319a045 22324618

[B47] Schaefer J. R. Leussler D. Rosin L. Pittrow D. Hepp T. (2014). Improved detection of paroxysmal atrial fibrillation utilizing a software-assisted electrocardiogram approach. *PLoS One* 9:e89328. 10.1371/journal.pone.0089328 24586692 PMC3938451

[B48] Seet R. C. Friedman P. A. Rabinstein A. A. (2011). Prolonged rhythm monitoring for the detection of occult paroxysmal atrial fibrillation in ischemic stroke of unknown cause. *Circulation* 124 477–486. 10.1161/CIRCULATIONAHA.111.029801 21788600

[B49] Shaffer F. Ginsberg J. P. (2017). An overview of heart rate variability metrics and norms. *Front. Public Health* 5:258. 10.3389/fpubh.2017.00258 29034226 PMC5624990

[B50] Singh-Manoux A. Fayosse A. Sabia S. Canonico M. Bobak M. Elbaz A. (2017). Atrial fibrillation as a risk factor for cognitive decline and dementia. *Eur. Heart J.* 33 1–7. 10.1093/eurheartj/ehx208 PMC5837240 28460139

[B51] Sirichand S. Killu A. M. Padmanabhan D. Hodge D. O. Chamberlain A. M. Brady P. A. (2017). Incidence of idiopathic ventricular arrhythmias: a population-based study. *Circ. Arrhythm. Electrophysiol.* 10:e004662. 10.1161/CIRCEP.116.004662 28183845 PMC5319731

[B52] Svennberg E. Engdahl J. Al-Khalili F. Friberg L. Frykman V. Rosenqvist M. (2015). Mass screening for untreated atrial fibrillation: the STROKESTOP study. *Circulation* 131 2176–2184. 10.1161/CIRCULATIONAHA.114.014343 25910800

[B53] Takabayashi K. Hamatani Y. Yamashita Y. Takagi D. Unoki T. Ishii M. (2015). Incidence of stroke or systemic embolism in paroxysmal versus sustained atrial fibrillation: the Fushimi atrial fibrillation registry. *Stroke* 46 3354–3361. 10.1161/STROKEAHA.115.010947 26514188

[B54] Thong T. McNames J. Aboy M. Goldstein B. (2004). Prediction of paroxysmal atrial fibrillation by analysis of atrial premature complexes. *IEEE Trans. Biomed. Eng.* 51 561–569. 15072210 10.1109/TBME.2003.821030

[B55] Vasan R. S. Zuo Y. Kalesan B. (2019). Divergent temporal trends in morbidity and mortality related to heart failure and atrial fibrillation: age, sex, race and geographic differences in the United States, 1991-2015. *J. Am. Heart Assoc.* 8:e010756. 10.1161/JAHA.118.010756 30955391 PMC6507208

[B56] Xin Y. Zhao Y. (2017). Paroxysmal atrial fibrillation recognition based on multi-scale wavelet α-entropy. *Biomed. Eng. Online* 16:121. 10.1186/s12938-017-0406-z 29061181 PMC5654099

[B57] Zhang Y. Wei S. Di Maria C. Liu C. (2016). Using Lempel–Ziv complexity to assess ECG signal quality. *J. Med. Biol. Eng*. 36 625–634. 10.1007/s40846-016-0165-5 27853413 PMC5083778

