# Supplementary material for: Prediction of Paroxysmal Atrial Fibrillation From Complexity Analysis of the Sinus Rhythm ECG: A Retrospective Case/Control Pilot Study
Source: Front Physiol. 2021 Feb 19;12:570705. doi: 10.3389/fphys.2021.570705 (PMC7933455; doi:10.3389/fphys.2021.570705)
Supplement: Supplementary file 1 [file Data_Sheet_1.docx]

### Supplement to the main paper

### *LZ76 complexity estimator source code and validation routine*

#include <iostream>

#include <string>

#include <cmath>

using namespace std;

// ---------------------------------

// see doi: 10.1103/physreva.36.842

int LempelZiv76(const string * s)

{

int i=0,k=1,l=1;

int k_max = 1;

int n = s->length();

int c = 1;

if (n<1)

{

cerr<<"Cannot calculate LZ76 of a NULL string\n" ;

exit(-1);

}

while (true)

{

if (s->at(i + k - 1) == s->at(l + k - 1))

{

k = k + 1;

if ((l + k) >= (n))

{

c = c + 1;

return c;

};

}

else

{

if (k > k_max) { k_max = k;}

i = i + 1;

if (i == l)

{

c = c + 1;

l = l + k_max;

if (l + 1 > n)

{

return c;

}

else

{

i = 0;

k = 1;

k_max = 1;

}

}

else

{ k = 1;}

}

}

return c;

}

double LZ76(const string * Data)

{

double _LZ76=LempelZiv76(Data)*log2(Data->length());

_LZ76=_LZ76/Data->length();

return _LZ76;

}

// ---------------------------------

int main(int , char *)

{

static string teststrings[]=

{ "01011010001101110010","1.51267",

// J. Amigo. Permutation Complexity in Dynamical Systems: Ordinal Patterns, Permutation Entropy and All That , p.3

"01010101010101010101","0.648289",

// source: http://www.dm.unibo.it/~farinell/tesi/Capitolo1.pdf , page 10

"01111000110110111010","1.51267",

// ibid.

"0011001010100111","1.5",

// Source: Blanc Schmidt Pezard Quantifying neural correlations using LZ complexity

"0000000100000000000000000100000000000000000010000000000000000010000000000000000010000000000000000001","0.46507",

// typical ECG beat-detection data

"11111111111111111111111111000000000011000000011111111111111111111111000000001100010000010000000","0.553251"

// typical ECG threshold-crossing data

};

cout <<" ** Self-test **"<<endl;

for (int i=0;i<6*2;i+=2)

{

cout<<endl<<teststrings[i]<<endl;

cout<<"Estimated LZ76 complexity:\t"<<LZ76(teststrings+i)<<endl<<"Expected outcome:\t\t"<<teststrings[i+1]<<endl;

}

return 0;

}

### *Expected program output*

*** Self-test **

01011010001101110010
Estimated LZ76 complexity:      1.51267
Expected outcome:               1.51267

01010101010101010101
Estimated LZ76 complexity:      0.648289
Expected outcome:               0.648289

01111000110110111010
Estimated LZ76 complexity:      1.51267
Expected outcome:               1.51267

0011001010100111
Estimated LZ76 complexity:      1.5
Expected outcome:               1.5

0000000100000000000000000100000000000000000010000000000000000010000000000000000010000000000000000001
Estimated LZ76 complexity:      0.46507
Expected outcome:               0.46507

11111111111111111111111111000000000011000000011111111111111111111111000000001100010000010000000
Estimated LZ76 complexity:      0.553251
Expected outcome:               0.553251
